# Is There Any Effect on Smell and Taste Functions with Levothyroxine Treatment in Subclinical Hypothyroidism?

**DOI:** 10.1371/journal.pone.0149979

**Published:** 2016-02-29

**Authors:** Kamil Baskoy, Seyid Ahmet Ay, Aytug Altundag, Onuralp Kurt, Murat Salihoglu, Ferhat Deniz, Hakan Tekeli, Arif Yonem, Thomas Hummel

**Affiliations:** 1 Department of Endocrinology and Metabolism, Haydarpaşa Training Hospital, Gulhane Military Medical School, Istanbul, Turkey; 2 Department of Otorhinolaryngology, Istanbul Surgery Hospital, Istanbul, Turkey; 3 Department of Otorhinolaryngology, Erzincan Military Hospital, Erzincan, Turkey; 4 Department of Otorhinolaryngology, Haydarpaşa Training Hospital, Gulhane Military Medical School, Istanbul, Turkey; 5 Department of Neurology, Haydarpaşa Training Hospital, Gulhane Military Medical School, Istanbul, Turkey; 6 Interdisciplinary Center "Smell & Taste", Department of Otorhinolaryngology, TU Dresden, Dresden, Germany; The University of Tokyo, JAPAN

## Abstract

Subclinical hypothyroidism has been accused for coronary heart disease, lipid metabolism disorders, neuropsychiatric disorders, infertility or pregnancy related problems with various strength of evidence. Currently there is insufficient knowledge about olfaction and taste functions in subclinical hypothyroidism. Aim of the present study is to investigate the degree of smell and taste dysfunction in patients with subclinical hypothyroidism. 28 subclinical hypothyroid patients, and 31 controls enrolled in the prospective study in Istanbul, Turkey. Subclinical hypothyroid patients were treated with L-thyroxine for 3 months. Psychophysiological olfactory testing was performed using odor dispensers similar to felt-tip pens (“Sniffin’ Sticks”, Burghart, Wedel, Germany). Taste function tests were made using "Taste Strips" (Burghart, Wedel, Germany) which are basically tastant adsorbed filter paper strip. Patients scored lower on psychophysical olfactory tests than controls (odor thresholds:8.1±1.0 vs 8.9±1.1, *p* = 0.007; odor discrimination:12.4±1.3 vs 13.1±0.9, *p* = 0.016; odor identification:13.1±0.9 vs 14.0±1.1, *p* = 0.001; TDI score: 33.8±2.4 vs 36.9±2.1, *p* = 0.001). In contrast, results from psychophysical gustatory tests showed only a decreased score for “bitter” in patients, but not for other tastes (5.9±1.8 vs 6.6±1.0, *p* = 0.045). Three month after onset of treatment olfactory test scores already indicated improvement (odor thresholds:8.1±1.0 vs 8.6±0.6, *p*<0.001; odor discrimination:12.4±1.31 vs 12.9±0.8, *p* = 0.011; odor identification:13.1±0.9 vs 13.9±0.8, *p*<0.001; TDI scores:33.8±2.4 vs 35.5±1.7, *p*<0.001) respectively. Taste functions did not differ between groups for sweet, salty and, sour tastes but bitter taste was improved after 3 months of thyroxin substitution (patients:5.9±1.8 vs 6.6±1.2, *p* = 0.045). Correlation of changes in smell and taste, with thyroid function test were also evaluated. TSH, fT4 were found have no correlation with smell and taste changes with treatment. However bitter taste found positively correlated with T3 with treatment(r: 0.445, p: 0.018). Subclinical hypothyroid patients exhibited a significantly decreased olfactory sensitivity; in addition, bitter taste was significantly affected. Most importantly, these deficits can be remedied on average within 3 months with adequate treatment.

## Introduction

Subclinical hypothyroidism is defined as a situation in which serum levels of free thyroxin (fT4) are normal and serum thyroid-stimulating hormone (TSH) levels are elevated [1]. Situations like recent levothyroxine adjustment which did not reach the steady state, recovery phase of severe illnesses, thyroiditis by different etiologies, external sources of TSH variance, presence of biochemically TSH mimicking markers were excluded from this definition [2].

Prevalence in adults was found in 4.3% of the U.S. population in National Health and Nutrition Examination Survey III [3] and ranged between 4–20% [4] in different studies. Prevalence of subclinical hypothyroidism is relatively high among elderly and women.

The conversion rate to overt hypothyroidism was found between 2% and 6% in subclinical hypothyroid patients. Higher TSH levels or presence of anti-thyroid peroxidase antibodies (Anti-TPO) increase the conversion risk [1]. Consequences of untreated subclinical hypothyroidism are controversial. Subclinical hypothyroidism has been accused for coronary heart disease, atherosclerosis, lipid metabolism disorders, neuropsychiatric disorders, infertility or pregnancy related problems with various strength of evidence [2,5]. It is claimed that these sequels are related to TSH levels [2].

Symptomatology is often unspecific; main complaints are fatigue, constipation, weight gain and cold intolerance [4]. Increased rate of cognitive dysfunction in subclinical hypothyroidism was shown in elderly population [6]. The most common diagnosis in subclinical hypothyroidism is Hashimoto Thyroiditis [2].

Currently there is insufficient knowledge about olfaction and taste functions in subclinical hypothyroidism, which represents a specific subgroup of hypothyroidism. Aim of the present study was to investigate the degree of smell and taste dysfunction in this group compared to healthy controls.

## Material and Methods

Study designed in prospective cross-sectional fashion and approved by the Clinical Research Ethics Committee of Haydarpasa Training Hospital (HNEAH-KAEK 2013/ KK/117). All subjects gave written informed consent and the institutional ethical committee approved the study protocol.

### Participants

Twenty eight subclinical hypothyroid patients, and 31, age, gender, education level matched participants enrolled in the study. All participants underwent complete otorhinolaryngological examination. Education level, demographic features were recorded. The control group was selected on the basis of the biochemical evidence of a normal thyroid function (TSH and fT_3_/fT_4_).

Patients having fT3/fT4 levels between local biochemistry laboratory's reference limits (fT3: 1.71–3.71 ng/dl, fT4: 0.7–1.48 ng/dl) and having higher values than upper reference limit for TSH (TSH>4.97 mU/I) were defined as subclinical hypothyroidism. Situations, causing temporal fluctuations in TSH and fT3/fT4 levels were excluded, like recovery from severe illnesses or presence of antibodies mimicking TSH. Exclusion criteria for both groups were previous history of olfactory dysfunction, history of upper respiratory tract infection in last three weeks, history of previous nasal surgery, chronic sinonasal diseases (chronic rhinosinusitis with or without presence of nasal polyposis, acute allergic rhinitis), usage of drugs known to affect smell or taste, severe head trauma, other endocrine disorders, psychiatric disorders, or current history of smoking.

Subclinical hypothyroid patients were treated with L-thyroxine for 3 months. Tests for smell, taste and endocrine function of patients and controls were recorded before and after treatment.

### Orthonasal Olfactory Testing

Psychophysiological olfactory testing was performed using odor dispensers similar to felt-tip pens (“Sniffin’ Sticks”, Burghart, Wedel, Germany) [7]. Validation studies for this test were already made [8,9]. The experimenter opens the pen and allows the patient to smell the odorant from 1–3 cm in front of the nostrils for three seconds. The test kit consists of 3 subtests: odor threshold, odor identification, and odor discrimination. The last two subtests are suprathreshold tests. The discrimination subtest consists of 16 triplets of pens, two of which contain the same odor while one is containing a different one. Patients are asked to identify the one pen which smells different. The identification test consists of 16 common odors; using a 4-alternative forced choice paradigm patients are asked to identify the respective odor from lists of 4 descriptors each. Threshold testing is made using 16 concentrations of phenyl-ethyl-alcohol presented within a single-staircase paradigm. Each concentration is presented together with 2 blanks; again, the subject’s task is to detect the smelling probe. Each subtest provides a maximum score of 16 points; the combined TDI score is the sum from the 3 scores of the 3 subtests (maximum 48).

### Taste Function Evaluation

Taste function tests were made using "Taste Strips" (Burghart, Wedel, Germany) which are basically tastant adsorbed filter paper strip [10]. The four basic tastants were presented in four concentrations each; sweet: 0.4, 0.2, 0.1, 0.05 g/ml sucrose; sour: 0.3, 0.165, 0.09, 0.05 g/ml citric acid; salty: 0.25, 0.1, 0.04, 0.016 g/ml sodium chloride; bitter: 0.006, 0.0024, 0.0009, 0.0004 g/ml quinine hydrochloride. Tastants were solved in distilled water. Before every application of a taste strips the mouth was rinsed with fresh water. All four tastants with four concentrations were applied to both sides of the anterior 1/3 of the tongue in increasing concentrations. Taste qualities were applied in a randomized fashion at each of the four levels of concentrations and alternating the side of presentation. With their tongue still extended, patients had to identify the taste from a list of four descriptors, i.e., sweet, sour, salty, and bitter (4 alterantive forced-choice). The number of correct answers were summated to obtain that side's taste score [11].

### Statistical Analysis

Statistical analyses were performed using SPSS software version 15 (SPSS Inc. Chicago, IL, USA). The variables were investigated using descriptive (histograms, probability plots) and analytical methods (Kolmogorov—Smirnov test) to determine whether or not they were normally distributed. Descriptive analyses were presented as means ± standard deviations for normally distributed variables. For non-normally distributed/ordinal variables descriptive statistics are presented with number of cases with percentage, medians and interquartile range (IQR). Differences between numeric variables of two groups were tested with independent samples Student’s t-test for continuous variables displaying normal distribution and Mann—Whitney U test for continuous variables not displaying normal distribution. Categorical variables (proportions) were compared by chi-square tests. Correlation analysis were made with Pearson’s and Spearman’s correlation analyses, respectively.

## Results

Our study included 31 controls and 28 untreated subclinical hypothyroid patients. Mean age was 29±8.5 years in the control group and 29.9±9.0 years in the study group. Groups did not differ significantly in terms of age, sex and education level (Table 1).

**Table 1:** Clinical Characteristics and Endocrine Parameters of Patients and Controls.

	Controls	Patients (before tx)	Patients (after tx)	p_1_ value	p_2_ value
Age*(years)	29 ± 8.54	29.93 ± 9.00	29.93 ± 9.00	0.686	-
Sex (female/male)	12/19	11/17	11/17	0.74	-
Education* (years)	11.48 ± 2.76	11.71 ± 3.53	11.71±3.53	0.78	-
TSH** (μIU/mL)	1.25 (0.9–1.73)	7.08 (6.38–9.44)	3.48 (2.63–4.12)	<0.001	<0.001
fT_4_* (ng/dL)	1.01 ± 0.11	0.98 ± 0.16	1.17 ± 0.18	0.506	<0.001
fT_3_* (pg/mL)	3.08 ± 0.50	3.02 ± 0.70	2.99±0.31	0.663	0.867
TPO-Ab** (IU/mL)	0.51 (0.2–0.8)	163.9 (8.5–801)	193.19 (5.1–888)	<0.001	0.311
Tg-Ab** (IU/mL)	0.66 (0.4–1.3)	10.35 (6.1–139)	13.66 (3–120)	0.021	0.396

tx, treatment; TSH, Thyroid-stimulating hormone; fT4, free thyroxine; fT3, free Triiodothyronine; TPO-Ab, Anti-thyroid peroxidase; Tg-Ab, Thyroglobulin antibody

*Values expressed in mean ± standard deviations.

**Values expressed in medians with percentiles. p_1_ value: controls versus patients (before tx). p_2_ value: Effects of after treatment with L-thyroxine on tests

**Fig 1:**
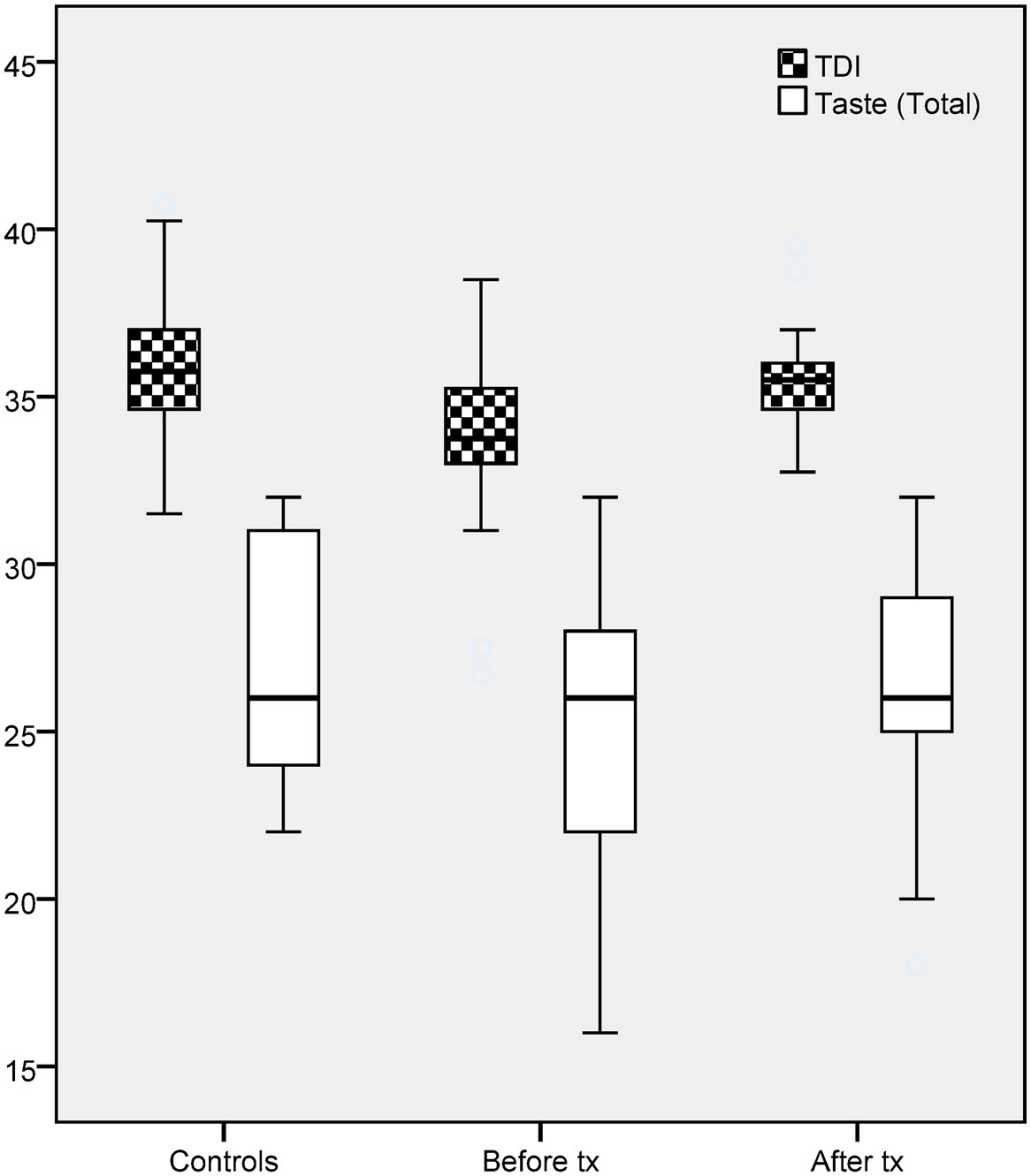
Comparison of TDI (Total score of Threshold + Discrimination + Identification) and taste total score in controls and subclinical hypothyroid patients (before and after treatment).

Patients scored lower on psychophysical olfactory tests than controls (odor thresholds: 8.1±1.0 vs 8.9±1.1, *p* = 0.007; odor discrimination: 12.4±1.3 vs 13.1±0.9, *p* = 0.016; odor identification: 13.1±0.9 vs 14.0±1.1, *p* = 0.001; TDI score: 33.8±2.4 vs 36.9±2.1, *p* = 0.001). In contrast, results from psychophysical gustatory tests showed only a decreased score for “bitter” in patients, but not for other tastes (5.9±1.8 vs 6.6±1.0, *p* = 0.045) (Table 2 and Fig 1).

**Table 2:** Olfactory and Taste results of Patients (before and after treatment) and Controls.

Test (mean±SD)	Controls	Patients (before tx)	Patients (after tx)	p_1_ value	p_2_ value
Threshold	8.89 ± 1.12	8.13 ± 0.97	8.63 ± 0.59	0.007	<0.001
Discrimination	13.06 ± 0.85	12.36 ± 1.31	12.93 ± 0.81	0.016	0.011
Identification	14.03 ± 1.05	14.03 ± 1.05	13.89 ± 0.79	0.001	<0.001
TDI	35.89 ± 2.07	33.77 ± 2.44	35.46 ± 1.72	0.001	<0.001
Bitter	6.64 ± 0.96	5.86 ± 1.80	6.58 ± 1.2	0.045	0.030
Sweet	6.58 ± 1.28	6.42 ± 1.76	6.5 ± 1.4	0.703	0.802
Salt	6.64 ± 1.20	6.58 ± 2.40	6.64 ± 1.22	0.814	0.745
Sour	6.58 ± 1.18	6.5 ± 1.18	6.64 ± 1.22	0.793	0.161
Total taste	26.52 ± 3.86	25.36 ± 4.32	26.36 ± 3.52	0.281	0.046

SD, standard deviations; tx, treatment. p_1_ value: controls vs patients (before tx). p_2_ value: Effects of after treatment with L-thyroxine on tests

Three month after onset of treatment olfactory test scores already indicated improvement (odor thresholds: 8.1±1.0 vs 8.6 ± 0.6, *p*<0.001; odor discrimination: 12.4±1.31 vs 12.9±0.8, *p* = 0.011; odor identification: 13.1±0.9 vs 13.9±0.8, *p*<0.001; TDI scores: 33.8±2.4 vs 35.5±1.7, *p*<0.001) respectively. Taste functions did not differ between groups for sweet, salty and, sour tastes but bitter taste was improved after 3 months of thyroxin substitution (patients: 5.9±1.8 vs 6.6±1.2, *p* = 0.045) (Table 2).

Correlation of changes in smell and taste, with thyroid function test were also evaluated. TSH, T4 were found have no correlation with smell and taste changes with treatment. However bitter taste found positively correlated with T3 with treatment (r: 0.445, p: 0.018) (Table 3).

**Table 3:** The correlations for differences in the changes of before and after treatment of olfactory-taste functions and thyroid function tests in subclinical hypothyroid patients.

	fT4		fT3		TSH	
Variable	r*	p*	r*	p*	r*	p*
Threshold	-0.334	0.082	-0.124	0.529	-0.145	0.46
Discrimination	0.109	0.58	0.135	0.495	0.014	0.942
Identification	-0.097	0.625	0.031	0.874	0.096	0.627
TDI	0.01	0.96	0.119	0.545	-0.085	0.667
Bitter	0.318	0.099	0.445	0.018	-0.012	0.952
Sweet	0.014	0.945	-0.235	0.229	-0.088	0.657
Salty	0.128	0.515	0.045	0.82	-0.164	0.403
Sour	0.052	0.795	-0.069	0.728	-0.155	0.432
Total Taste	0.29	0.134	0.112	0.571	-0.148	0.451

fT4, free thyroxine; fT3, free Triiodothyronine; TSH, Thyroid-stimulating hormone.

*Pearson Corelation analysis was used

## Discussion

The present studies revealed that patients with hypothyroidism score lower on olfactory tests and tests for bitter sensitivity compared to healthy controls, and that olfactory function and bitter sensitivity increase following 3 months of treatment with thyroid hormones. Importantly, the present study focused on patients with subclinical hypothyroidism whereas previous studies looked at clinically manifest hypothyrodism only.

In the study by McConnell [12] et al. smell and taste function was tested in 18 overt hypothyroid patients before and after treatment. Smell and taste tests were made with interrogation with the patients and similar testing methods which we have used. Dysosmia was seen on 39% of patients while dysgeusia was 50% before treatment. This study indicates that taste and smell deficits are common in hypothyroid patients and these deficits could be reversed with treatment.

It is still controversial which mechanisms are playing a role for taste and smell loss in hypothyroidism patients. Different studies revealed that hypothyroidism has effects at multiple points of the gustatory and olfactory perceptual pathways. Receptors, central olfactory and gustatory areas, high order cognitive systems are all targets of hypothyroidism. The currently investigated effects could be related to lesions at one or several levels. Using immunohistochemistry Dhong et al. [13] evaluated hypothyroidism-related changes at the level of the olfactory epithelium of adult rats and found a Propyl Thio-Urasil (PTU)-exposure related decrease in the maturation of olfactory receptor neurons. However total receptor count and density, olfactory epithelium surface area and thickness were found not to differ in relation to exposition with PTU. Paternostro et al. [14] assessed effects of hypothyroidism on the olfactory epithelium in newborn rats during their development and also found a decreased maturation rate in olfactory receptor cells; surface epithelium thickness was not changed by incremental exposure time with PTU.

Using PTU Zhang [15] et al investigated the influence of hypothyroidism, from the neonatal period to adulthood, on the neurogenesis of rats considering the idea that important developmental processes in the dentate gyrus, olfactory bulb, and cerebellum are postnatally still going on. Results of thyroid hormone deprivation on these specific cells were investigated; the authors found a loss in total brain weight, reduced cell count in the dentate gyrus, and reduced hippocampal volume. Because the olfactory bulb volume was found to be unaffected it may be speculated that the cause for hypothyroidism-associated hyposmia may be due to functional changes at the receptors or in the upper olfaction areas of the brain.

Overt hypothyroidism has well known effects upon cognitive functions [4,16]. Bajaj [6] et al. was searched for cognitive functions in subclinical hypothyroid patients. This study revealed that increasing levels of TSH correlate with the decline in cognitive functions. Animal studies revealed global affection of brain from hypothyroidism in terms of weight loss and cognitive decline. The olfactory system is expected to be influenced as a part of neurologic system. Hovewer, Brosvic [17] et al. investigated behavioral differences in hypothyroid and control rats; they found no significant differences in odor detection performance between the two groups.

Sensitivity for “bitter” was significantly decreased in hypothyroid patients compared to controls. Importantly, this deficit was remedied during the 3-months treatment period. Recent work by Clark et al. [18] indicated that tasting of bitter compounds would modulate thyrocyte function and T3/T4 production. Thus, the present findings could be the result of a complex network between taste and thyroid functions.

Definition of subclinical hypothyroidism requires normal levels of T3/T4 and increased TSH. Treatment of subclinical hypothyroidism doesn't change levels of free T3 and T4 levels, reduces TSH levels to normal interval. Günbey et al. [19] investigated olfactory function in primary hypothyroid patients and found significantly lower scores in the hypothyroid group and a positive correlation between all threshold, discrimination and identification scores and free T3 levels. Accordingly, we also looked for a similar relation between olfaction and thyroid function tests in subclinical hypothyroid patients but did not find a positive correlations. Regarding changed fT3 levels in the normal interval of our results, expected correlation wouldn't be occured. In this context our results has no contradiction with previous study.

## Conclusion

Subclinical hypothyroid patients exhibited a significantly decreased olfactory sensitivity; in addition, bitter taste was significantly affected. Most importantly, these deficits can be remedied on average within 3 months with adequate treatment.

## Supporting Information

S1 Data Supporting information file. (XLS)
